# Reviewed article: Silva SN, Saliba MF, Ribeiro LR, Cota GF. Miltefosine implementation for treatment of cutaneous leishmaniasis: access indicators in the state of Minas Gerais, Brazil (2021-2024). Epidemiol Serv Saude. 2025:34;e20240690.

**DOI:** 10.1590/S2237-96222025v34e20240690.a

**Published:** 2025-06-20

**Authors:** Tayanny Margarida Biase

**Affiliations:** Universidade Estadual de Campinas, School Pharmaceutical Science

**Table:** 

Rating	Excellent	Good	Average	Below average	Poor
Interest		✓			
Quality			✓		
Originality			✓		
Overall		✓			

**Table:** 

Questionnaire	Yes	No	Not applicable
Does the manuscript contain new and significant information to justify publication?	✓		
Does the Abstract (Summary) clearly and accurately describe the content of the article?	✓		
Is the problem significant and concisely stated?	✓		
Are the methods described comprehensively?	✓		
Are the interpretations and conclusions justified by the results?	✓		
Is adequate reference made to other work in the field?	✓		

## Manuscript Structure

Length of article is: Adequate

Number of tables is: Adequate

Number of figures is: Adequate

Please state any conflict(s) of interest that you have in relation to the review of this paper (state “none” if this is not applicable): None

## Comments

Explique o significado de cada sigla na primeira vez que ela aparecer no texto. Por exemplo, na sessão de discussão, linha 51, descreva a sigla “JBI”.

O manuscrito precisa ser revisado para corrigir erros gramaticais e ortográficos. Revisar espaço e a falta de espaço entre palavras. A concordância das frases precisa ser verificada.

Assegure-se de que as citações estejam de acordo com o estilo da revista. Em alguns pontos do texto as referências são citadas com e sem espaço após a última palavra. Revise todo o texto e siga o padrão da revista.

Sugiro incluir na Tabela 2 que o tempo de análise foi em “dias”.

Métodos

Descreva os métodos com mais detalhes, sugiro seguir o checklist STROBE. Descreva de forma clara os tópicos contexto e delineamento, parece haver repetição entre eles. No tópico participantes e variáveis, para cada variável forneça as fontes de dados e mensuração. Por exemplo, para varável sexo (masculino, feminino), tipo de leishmaniose (descreva quais os tipos avaliados).

O deslocamento de pacientes para outras macrorregionais e o impacto disso não são mencionados nos métodos, mas aparecem nos resultados. Embora a análise seja relevante, sugiro incluir no delineamento para maior consistência.

Resultados

Os resultados estão de acordo com os métodos descritos, mas há algumas análises adicionais que surgem nos resultados e não foram previstas inicialmente (como o deslocamento dos pacientes). Embora isso enriqueça o estudo, a inclusão prévia desses aspectos nos métodos teria dado maior robustez e previsibilidade à análise.

Discussão

Inclua um parágrafo com as limitações do estudo na sessão de discussão (ausência de controle sobre a qualidade dos dados; não consideração de fatores contextuais, possíveis barreiras, como disponibilidade de profissionais capacitados, infraestrutura, ou questões logísticas; dependência de dados administrativos, os dados podem não capturar aspectos qualitativos da experiência dos pacientes e profissionais, como satisfação, dificuldades ou percepção do tratamento).

